# Mendelian randomization analyses in ocular disease: a powerful approach to causal inference with human genetic data

**DOI:** 10.1186/s12967-022-03822-9

**Published:** 2022-12-26

**Authors:** Jiaxin Li, Cong Li, Yu Huang, Peng Guan, Desheng Huang, Honghua Yu, Xiaohong Yang, Lei Liu

**Affiliations:** 1 Department of Epidemiology, School of Public Health, China Medical University, Shenyang, Liaoning China; 2 Guangdong Eye Institute, Department of Ophthalmology, Guangdong Provincial People’s Hospital, Guangdong Academy of Medical Sciences, Guangzhou, 510080 China; 3 Guangdong Cardiovascular Institute, Guangdong Provincial People’s Hospital, Guangdong Academy of Medical Sciences, Guangzhou, China; 4 Department of Mathematics, School of Fundamental Sciences, China Medical University, Shenyang, Liaoning China

**Keywords:** Causality, Eye disease, Instrumental variables, Mendelian randomization

## Abstract

Ophthalmic epidemiology is concerned with the prevalence, distribution and other factors relating to human eye disease. While observational studies cannot avoid confounding factors from interventions, human eye composition and structure are unique, thus, eye disease pathogenesis, which greatly impairs quality of life and visual health, remains to be fully explored. Notwithstanding, inheritance has had a vital role in ophthalmic disease. Mendelian randomization (MR) is an emerging method that uses genetic variations as instrumental variables (IVs) to avoid confounders and reverse causality issues; it reveals causal relationships between exposure and a range of eyes disorders. Thus far, many MR studies have identified potentially causal associations between lifestyles or biological exposures and eye diseases, thus providing opportunities for further mechanistic research, and interventional development. However, MR results/data must be interpreted based on comprehensive evidence, whereas MR applications in ophthalmic epidemiology have some limitations worth exploring. Here, we review key principles, assumptions and MR methods, summarise contemporary evidence from MR studies on eye disease and provide new ideas uncovering aetiology in ophthalmology.

**Supplementary Information:**

The online version contains supplementary material available at 10.1186/s12967-022-03822-9.

## Introduction

Causal inference plays a central role in both epidemiological and clinical investigations. Understanding the underlying aetiology is implicit for identifying disease prevention and treatment opportunities. Conventionally, observational ophthalmic epidemiology or clinical studies have identified causal inferences between exposures (lifestyle or biomarkers) and outcomes (ocular disorders) of interest. However, due to different challenges such as unmeasured and residual confounders, where preclinical or disease status influences exposure rather than vice versa, study findings are often controversial [1]. The prospective ophthalmic cohort model is the most reliable causal test assessing the impact of exposures on outcomes. Furthermore, randomised controlled trials (RCTs) also offer high level evidence for causality. Nevertheless, cohort studies or RCTs that elucidate disease outcomes are often unfeasible due to strict quality control, comprehensive design, long-term follow-up, pleiotropic effects of the intervention, ethical issues and a lack of compliance.

**Fig. 1:**
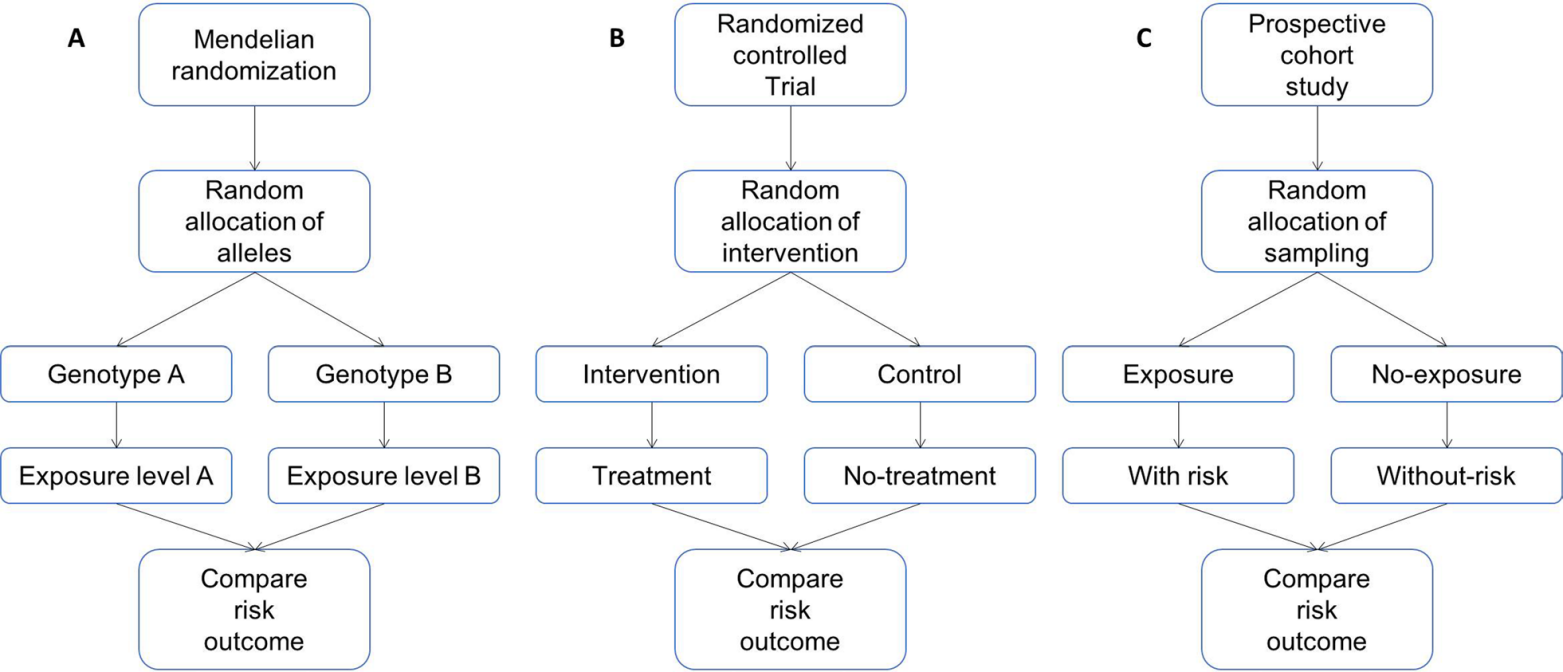
The parallel relationship between the design flow charts of the three studies. **A** Mendelian randomization (MR): It uses randomly assigned the genetic variant to be equivalent to the subgroups of randomized controlled trial (RCT). **B** RCT: It also divides the population into different subgroups according to the way of random distribution, so that the intervention between groups is different. **C** Prospective cohort study: It is an observational research method that divides a group of cohorts into exposure group and non-exposure group according to whether they are exposed to a certain research factor to judge whether there is a correlation between the exposure factor and disease

Recently, Mendelian randomization (MR) analysis has gradually entered the field of researchers with its new perspective to infer the causal relationship between exposures and outcomes. Based on observational epidemiology, it mainly uses natural variable genes that are not affected by acquired confounding factors as instrumental variables (IVs) for analysis to achieve random assignment to infer potential environmental factors that can lead to disease [2]. Observational studies develop 'Berkson's bias' when investigating associations between exposures and outcomes, resulting in spurious directions of associations by excluding people who were exposed and at lower risk (outcomes) [2]. MR is a term that refers to the fusion of gametes, following Mendel's law of inheritance" parental alleles are randomly assigned to the offspring", genetic variation is unaffected by traditional confounding factors, such as environmental exposure, socioeconomic status, behavioural factors or biomarkers [3]. In diploid organisms, two copies of each allele are normally inherited from the parents, and their associations with disease are chronologically rational. Compared to traditional observational study, MR can overcome confounding issues and reverse causality[4]. With the development of particular research technologies (e.g., large genome-wide association studies (GWAS), epigenetics and metabolomics), MR analysis has been widely used for causal inference; it not only verifies conclusive causality (e.g., glucose levels in normo-glycaemic and higher ranges exerting genetic causal effects on the risk of micro- and macro-vascular disease [5]), but also provides more reliable conclusions for long-standing controversial causal association issues (e.g., some metabolic marker levels are not associated with choroidal neovascularisation [6]). MR combines observational study designs with RCT designs (Fig. 1). The similarities with RCT designs are reflected by randomised subgroups in simulated RCTs which reflect different genetic variation to exposure factors (e.g., vitamin D supplements may increase the risk of Behçet's disease (BD) [7]). Moreover, MR research leverages exposure and outcome data from observational research, which greatly reduces costs. Specifically, MR approaches assess the long-term consequences of an exposure, such as lifestyle or a biomarker, whereas cohort and RCTs typically focus on short-term assessments due to high costs.

In the last decade, MR analysis has become a popular tool for estimating the causality of an exposure on an ocular outcome. Here, we review the contemporary, state-of-the-art evidence for MR and its associated analytic approaches, further summarise evidence from MR-oriented ophthalmology studies and evaluate potential challenges and prospects for this novel approach.

### The MR framework

Epidemiologists have long sought an "ideal" method providing clear causality and ignoring potential confounding factors. Over the last three decades, this ideal has turned into reality when IVs were identified [8]. Initially, IV-based techniques were used in econometrics, such as the simultaneous regression equations, structural equations and two-stage least squares (2SLS) [9]. The earliest application of IVs, in modern medicine as we know it, was by Permutt and Hebel in 1989 who studied the relationship between maternal smoking and low birth weight incidences in infants [10]. After this, Greenland [11] comprehensively described the IV methods and its development. MR is advantageous in that it obtains the estimation of causal effect (X–Y) by using genetic IVs (Z) to avoids confounder interference (U). The following three assumptions are the basic assumptions that need to be met for IVs (Additional file 1: Fig. S1):

i. IV Z is robustly associated with exposure, X
ii. The variable Z is independent of an unmeasured confounding factor, U;
iii. The IV Z has an effect on outcome Y mediated solely through exposure X.

But, not all genetic variations can be selected to assess the causal association between the exposure and the outcome. The primary and critical task in MR research has always been to find suitable IVs. The potential limitations required to ensure compliance with the three assumptions are as follows: (1) If genetic variant used as IV is in linkage disequilibrium (non-random correlation between alleles at different loci), another genetic variant in linkage disequilibrium may be associated with confounders, a mediator between IV and exposure (equivalent to vertical pleiotropy), or directly related to the outcome, therefore leading to a violation of the assumptions ii or iii. In this case, the correlation between known confounding factors and IVs should be examined carefully to avoid it. (2) The IVs satisfy hypothesis i, but have weak correlation with exposure, should be investigated in large sample research [12]. (3) The selected sample population must be homogeneous to avoid the confounding of population differences. (4) Pleiotropy is the phenomenon in which one gene locus affects multiple phenotypes, including horizontal and vertical [13].The horizontal pleiotropy refers to genes directly affect multiple phenotypes, and vertical pleiotropy means genes use one phenotype as an intermediary to influence another phenotype. Of note, the horizontal pleiotropy violates the basic hypothesis of MR and might conclude misleading causal estimation. Subsequent sensitivity analysis (e.g. gene pleiotropy test, heterogeneity test and leave-one-out method) can be used to evaluate the robustness of the results and correct for bias.

### The design of MR

The early basic MR design can only solve whether there is a causal relationship between exposure and outcome, stay at the logical level, and cannot get the estimation of X causal effect. With the continuous improvement of MR design, two common MR research strategies are formed: one-sample MR and two-sample MR, also known as standard MR. Standard MR optimises the basic MR concept by constructing a quadratic regression model and calculating the estimated value of the effect of X–Y (Additional file 1: Fig. S2). There are also differences in the way of causal estimation in these two MR methods. One-sample MR relies on 2SLS to evaluate causal estimates, using the X value predicted by IV as the independent variable, and performing least squares regression with the outcome Y as the dependent variable. The magnitude of the predicted X value depends on the closeness between the IVs and the exposure, and weak instrumental bias can easily affect the causal estimate. However, the two-sample MR can calculate the causal effect value through two independent GWAS databases, not through the regression of X–Y, but according to the ratio of the regression coefficient of G- Y and G-X. As can be seen from the definition names assigned, the biggest difference between the two is that the subjects of one-sample MR are from a single population; the subjects of two-sample MR are from two independent samples of the same population. Research development of large-scale GWAS has made two samples MR data acquisition becomes more convenient, in-depth study of field and more widely. To adequately explore causality, MR methods are continuously developing (Additional file 1: Fig. S3). Two-step MR and bidirectional MR need to be analyzed twice in two samples. Two-step MR involves mediators which identify potential pathogenesis in the causal relationship between exposure and outcome, while bidirectional MR clarifies the direction of the causal correlation. Similarly, pleiotropic genetic variations are associated with homogeneous multi-classification exposure factors, thus a multivariable MR approach is appropriate.

### MR theory

The fundamental reason for the widespread popularity of MR stems from the steady stream of sharable genetic data provided by large-scale GWAS studies conducted among collaborating groups around the world. In addition, the continuous improvement of MR design strategy can also make causal inference using multi-IVs. Several MR analyses based on summary data using multi-IVs are commonly used (Table 1).

**Table 1:** Comparisons of the three Mendelian randomization (MR) methods

	IVW method	MR-Egger method	GSMR method
Theory	The weighted linear regression of $\widehat{\beta }$_YC_ and $\widehat{\beta }$_XG,_ as well as the forced intercept term is equal to zero	The weighted linear regression of $\widehat{\beta }$_YC_ and $\widehat{\beta }$_XG_	Generalised least squares method of $\widehat{\beta }$_XY_
R package function	MR-IVW function	MR-Egger function	GSMR function
Application conditions	IVs are satisfied with a correlation hypothesis, independence hypothesis and exclusivity hypothesis	IVs are satisfied with a correlation hypothesis and independence hypothesis	IVs are satisfied with a correlation hypothesis, independence hypothesis and exclusivity hypothesis
Advantages	i) When IVs lose pleiotropy, the estimation accuracy is high and the power of a test is high	i) Unbiased estimations are obtained when IVs display pleiotropy ii) The average pleiotropy can be equalised by the intercept term iii) Sensitivity analysis can be performed	i) When IVs lose pleiotropy, the estimation accuracy is high and the power of a test is high ii) Can be tested by heterogeneity in dependent instruments (HEIDI) whether IVs have pleiotropy
Disadvantages	i) When IVs display pleiotropy, the estimation is biased	i) The estimation is biased, and the false positive error is inflated ii) When there is no pleiotropy, the power of a test is lower than IVW and GSMR	i) When IVs display pleiotropy, the estimation is biased
limitations	Screen out the pleiotropic IVs	Require all IVs’ direction is the same	HEIDI delete pleiotropic IVs
Summary data	Homogenous population or the population after correcting the group structure		

*IVW* inverse-variance-weighted, *GSMR* generalised summary Mendelian randomization, *MR-Egger* Mendelian randomization-Egger, *IVs* instrumental variables

### The inverse variance weighted (IVW) method

The IVW method was proposed by Burgess et al*.* and is used in multi-instrumental variable MR analysis [14, 15]. Herein, we define $G = \left\{ {G_{1} ,\,G_{2} ,G_{3} ...G_{J} } \right\}$ as IVs, X as an exposure factor and Y as an outcome. We assume the j-th VI ($G_{j}$) has an impact on exposure factor and outcome, as $\widehat{\beta }xG_{j}$ and $\widehat{\beta }yG_{j}$, respectively. Furthermore, their standard errors are $\sigma_{{\widehat{\beta }xG_{j} }}$ and $\sigma_{{\widehat{\beta }yG_{j} }}$, respectively. The corresponding causal effect value is obtained by the ratio method: $\widehat{\beta }xy_{j} = \widehat{\beta }xG_{j} /\widehat{\beta }yG_{j}$

After this, we calculate the causal effect value between exposure factor and outcome:

1 $${{\widehat{\beta }}_{IVW}}={\frac{{\sum_{j=1}^{J}}{{\widehat{\beta }}_{Y{G}_{j}}}{{\widehat{\beta }}_{X{G}_{j}}}/{{\sigma }_{{\widehat{\beta }}_{Y{G}_{j}}}^{2}}}{{{\sum }_{\mathrm{j}=1}^{J}}{{\widehat{\beta 2}}_{X{G}_{j}}}{{\sigma }_{{\widehat{\beta }}_{Y{G}_{j}}}^{-2}}}}$$

The asymptotic variance of this IV estimator is given by the formula:

2 $${\sigma }_{{\widehat{\beta }}_{IV{W}_{-corr}}}=\sqrt{{\left({{\widehat{\beta }{^{\prime}} }_{XG}\Omega }^{-1}{\widehat{\beta }}_{XG}\right)}^{-1}}$$

where ${\sigma }_{{\widehat{\beta }}_{Y{G}_{J}}}^{-2}$*,* is defined as a weight, and weighted linear regression is used to force the intercept term of linear regression to be 0, whereas:

3 $${\widehat{\beta }}_{Y{G}_{j}}={\beta }_{IVW}{\widehat{\beta }}_{X{G}_{j}}+{\xi }_{Ij}, {\xi }_{Ij}\sim N\left(0, {\sigma }^{2}{\sigma }_{{\widehat{\beta }}_{Y{G}_{j}}}^{-2}\right)$$

Then, the correspondence causal effect is:

$${{\widehat{\beta }}_{IV{W}_{-}Corr}}={{\left({{\widehat{\beta }{^{\prime}} }_{XG}\Omega }^{-1}{\widehat{\beta }}_{XG}\right)}^{-1}}{{\widehat{\beta }{^{\prime}} }_{XG}{\Omega }^{-1}}{{\widehat{\beta }}_{\mathrm{YG}}}$$

### MR Egger regression

MR Egger regression is an update version of the IVW method where multi-effects exist between IVs. The formula is:

4 $${\widehat{\beta }}_{Y{G}_{j}}={\beta }_{0E}+{\beta }_{1E}{\widehat{\beta }}_{X{G}_{j}}+{\xi }_{Ej}, {\xi }_{Ej}\sim N(0,{\sigma }^{2}{\sigma }_{{\widehat{\beta }}_{Y{G}_{j}}}^{ 2})$$

### Generalised summary data based MR (GSMR)

GSMR is based on summary data based MR (SMR). The estimated effect of IV is ${\widehat{\beta }}_{\mathrm{XY}}=\left\{{\widehat{\beta }}_{X{Y}_{1}} , {\widehat{\beta }}_{X{Y}_{2}} , \cdot \cdot \cdot {\widehat{\beta }}_{X{Y}_{\mathrm{j}}}\right\}$ and the variance–covariance matrix is:

5 $$\mathrm{COV}\left({\widehat{\beta }}_{X{Y}_{i}},{\widehat{\beta }}_{X{Y}_{j}}\right)\approx\, \frac{r}{{\beta }_{X{G}_{i}}{\beta }_{X{G}_{j}}}\sqrt{{\sigma }_{{\widehat{\beta }}_{Y{G}_{i}}}^{2}{\sigma }_{{\widehat{\beta }}_{Y{G}_{\mathrm{j}}}}^{2}}+{\beta }_{X{Y}_{i}}{\beta }_{X{Y}_{j}}\left[\frac{r\sqrt{{\sigma }_{{\widehat{\beta }}_{X{G}_{i}}}^{2}{\sigma }_{{\widehat{\beta }}_{X{G}_{j}}}^{2}}}{{\beta }_{X{G}_{i}}{\beta }_{X{G}_{j}}}-\frac{\sqrt{{\sigma }_{{\widehat{\beta }}_{X{G}_{i}}}^{2}{\sigma }_{{\widehat{\beta }}_{X{G}_{j}}}^{2}}}{{\beta }_{X{G}_{i}}^{2}{\beta }_{X{G}_{j}}^{2}}\right]$$

6 $${\sigma }_{{\widehat{\beta }}_{X{Y}_{i}}}={{\widehat{\beta }}^{2}}_{X{Y}_{i}}\left[\frac{{\sigma }_{{\widehat{\beta }}_{X{G}_{i}}}^{2}}{{\beta }_{X{G}_{i}}^{2}}+\frac{{\sigma }_{{\widehat{\beta }}_{Y{G}_{i}}}^{2}}{{\beta }_{\mathrm{Y}{G}_{i}}^{2}}-\frac{{\left({\sigma }_{{\widehat{\beta }}_{X{G}_{i}}}^{2}\right)}^{2}}{{\beta }_{X{G}_{i}}^{4}}\right]$$

## MR ophthalmology studies

Many studies have explored, confirmed and quantified causal relationships between potential exposures and the risk of eye disease. To investigate the application of MR to eye disease, we systemically searched PubMed, EMBASE and Web of Science databases for studies using the search keywords, “Mendelian randomization” with any outcomes related to “eye OR ocular OR ophthalmology”. Database were searched from inception to 1^st^ May 2021.

Inclusion criteria: (a) the exposure of interest was a lifestyle or biological factor related to eye disease, (b) the outcome was any eye disease and (c) genetic variants or genetic risk scores (GRS) were proposed as IVs to analyse the effects of exposures on outcomes. We excluded articles that did not use MR to explore causal associations in ophthalmology. Also, we excluded studies where ophthalmic conditions were identified as exposures rather than outcomes. Finally, 66 studies were included and independently reviewed by two co-authors (JXL & LL); any disagreements were resolved by discussion. These studies provided information on factors associated with eye disease aetiology (Table 2). Due to rapid developments in ophthalmology genetics research, we envisage MR will become widely used and help identify both modifiable and non-modifiable causes of disease.

**Table 2:** Mendelian randomization studies in ophthalmology

Study	Exposure	Outcome	Source	N	IVs	MR method	OR/beta (95% CI/SE)
Lim et al. 2009 [20]	BMI	Any, Cortical, Nuclear, PSC	Singapore Malay Eye Study	3000	10 SNPs	One-sample MR	Any: 1.17 (1.00, 1.37) Cortical: 1.33 (1.11, 1.58) Nuclear: 1.00 (0.87, 1.16) PSC: 0.86 (0.70, 1.07)
Sharma et al. 2013 [28]	CFH	AMD	PGIMER	176	rs1061170	One-sample MR	NA
Shen et al. 2016 [100]	T2D; Pancreatic β-cell function	Glaucoma	GERA	80,953	39 SNPs	Separate-sample genetic instrumental variable analyses	T2D: 2.53 (1.04, 6.11) Pancreatic β-cell function: 5.26 (1.75, 15.85)
Li et al. 2016 [172]	SBP DBP MAP	CRAE CRVE	A multi-ethnic cohort in Singapore	6258	GRS	Bidirectional MR	SBP-CRAE: − 1.3 (− 2.8, 0.3) SBP-CRVE: − 1.0 (− 3.7, 1.7) DBP-CRAE: − 4.5 (− 7.9, -1.0) DBP-CRVE: − 2.3 (− 7.4, 2.7) MAP-CEAE: − 2.6 (− 5.2, 0.01) MAP-CRVE: 0.2 (− 3.6, 4.0)
Cuellar-Partida et al. 2016 [57]	Education	Myopia	AREDS BMSE KORA	5649	PRS	TSLS	AREDS: − 1.33 (0.42) BMSE: − 0.87 (0.71) KORA: − 0.64 (0.45)
Cuellar-Partida et al. 2017 [64]	Serum vitamin D level	Myopia	CREAM	37,382; 8376	4 SNPs	One-sample MR	Caucasians: -0.02 (-0.09, 0.04) Asians: 0.01 (-0.17, -0.19)
Sobrin et al. 2017 [141]	Plasma lipid levels	Any DR + severe DR	18 GWASs on DR; GLGC + AGEN	2969 + 4096; 1277 + 3980	157 + 51 SNPs	Two-sample MR	Severe DR: HDL-C: 0.98 (0.74, 1.31) LDL-C: 0.95 (0.39, 2.36) TG: 0.84 (0.33, 2.12) Any DR: HDL-C: 0.91 (0.67, 1.23) LDL-C: 0.95 (0.91, 6.87) TG: 1.00 (0.86, 1.15)
Burgess et al., 2017 [47]	HDL-C	AMD	GLGC + IAMDGC	16,144 + 17,832	86 SNPs	Two-sample MR	1.22 (1.03, 1.44)
Fan et al. 2017 [48]	HDL-C	Advanced AMD	IAMDCG + GAMA	16,144 + 17,832; 2219 + 5275	96 SNPs	Two-sample MR	Three models: 1.30 (1.09, 1.55) 1.21 (1.11, 1.31) 1.17 (1.07, 1.29)
Mountjoy et al. 2018 [60]	Education	Myopia	23andMe; SSGAC; UKBB	67,798	44SNPs 69SNPs	Bidirectional, two-sample MR	Time spent in education-RE: − 0.270 (− 0.368, 0.173) RE-Time spent in education: -0.008 (-0.041, 0.025)
Hysi et al. 2019 [103]	O-methylasorbate levels	IOP	EPIC UKBB	103,382	13 SNPs	Two-sample MR	UKBB: − 0.696 (0.304) EPIC: − 3.219 (1.371)
Tan et al. 2019 [21]	obesity	cortical, nuclear, PSC	BMES	3654	rs 9,939,609	Two-stage MR	Cortical: 1.31 (0.86, 1.99) Nuclear: 1.04 (0.65, 1.68) PSC: 1.42 (0.75, 2.69)
Wood et al. 2019 [53]	RE	AMD	UKBB; IAMDGC	16,144 + 17,832	126 SNPs	Two-sample MR	1.080 (1.021, 1.142)
Tideman et al. 2019 [69]	Birth height BW	AL; CR; AL/CR	A population-based prospective birth cohort	3880	GRS	Two-stage least square method	Height-AL: 0.046 (0.016) BW-AL: − 0.023 (0.011); Height-CR: 0.026 (0.006) BW-CR: − 0.013 (0.004); Height-AL/CR: − 0.004 (0.002) BW-AL/CR: 0.002 (0.001)
Emanuelsson et al. 2019 [150]	LDL-C	retinopathy	CCHS + CGPS; GLGC + UKBB	109,102 408,455	11SNPs 45SNPs	One-sample MR; two-sample MR	One-sample MR: 1.06 (0.24–4.58) Two-sample MR: 0.83 (0.44, 1.56)
Emanuelsson et al. 2020 [5]	Glucose levels	retinopathy	CCHS + CGPS; MAGIC + UKBB	114,994 452,264	7SNPs 26SNPs	One-sample MR; Two-sample MR	One-sample MR: 2.01 (1.18, 3.41) Two-sample MR: 4.55 (2.26, 9.15)
Plotnikov et al. 2020 [73]	BW	RE	UKBB	39,658	Allele score	One-sample MR	0.28 (0.05, 0.52)
Xu et al. 2020 [107]	LDL-C HDL-C TG	POAG	GLGC; UKBB	463,010	185 SNPs	Two-sample MR	LDL-C: − 0.00026 (− 0.00062, 0.00011) HDL-C: 0.00023 (− 0.00015, 0.00061) TG: -0.00028(− 0.00071, 0.00015)
Simcoe et al. 2020 [123]	IOPcc	CH; CRF	UKBB	108,480	144SNPs 166SNPs 99SNPs 96SNPs	One-sample MR	CH-IOPcc:-0.128 (0.024) CRF-IOPcc: 0.31 (0.021); IOPcc-CH: -0.154 (0.009); IOPcc-CRF: 0.137 (0.01)
Choquet et al. 2020 [132]	CCT	POAG	IGGC; GERA	44,039	26SNPs	Two-sample MR	1.00 (1.00)
Huang et al. 2020 [147]	APN	DR	TWB; CMUH	438 + 813	47SNPs 16SNPs 5SNPs	Two-sample MR	GRS_ALL_: 0.61 (0.10, 1.13) GRS_Limited_: 0.57 (− 0.06, 1.21) GRS_APN_: 0.20 (-0.46, 0.85)
Han et al. 2020 [86]	RE IOP	RRD	UKBB	4257 + 39,181	224SNPs 99SNPs	Two-Sample MR; multivariable MR	RE: 0.72 (0.69, 0.76) IOP: 1.08 (1.03, 1.14)
Han et al. 2020 [30]	CPR	AMD	UKBB; IAMDGC	12,711 + 14,590	526SNPs	Two-sample MR	1.31 (1.19, 1.44)
Han et al. 2021 [6]	Eight major serum lipid biomarkers	Advanced AMD; Intermediate AMD	UKBB; IAMDGC	12,711 + 5336 + 14,590	64-407SNPs	Two-sample MR	HDL-C-Advanced AMD: 1.19 (1.07, 1.33) LDL-C-Advanced AMD: 0.87 (0.76, 1.00) TG-Advanced AMD: 0.81 (0.72, 0.90) TG-Intermediate AMD: 0.74 (0.66, 0.83) Lp(a)-Advanced AMD: 1.00 (0.89, 1.12); Lp(a)-Intermediate AMD: 0.96 (0.85, 1.09) et al
Patasova et al. 2021 [77]	Medication-Taking	RE	UKBB	102,318	447SNPs	One-sample MR	IOP-lowering drugs-RE: − 0.043 (− 0.064, − 0.022) Chronic multisite pain-RE: 0.210 (0.115, 0.287) et al
Kim et al. 2021 [127]	Coffee intake	IOP	UKBB	92,699	8SNPs	Two-sample MR	0.12 (-0.14, 0.39)
Nusinovici et al. 2021 [110]	HDL-3	POAG	SEED; IGGC	10,033	4SNPs	Two-sample MR	0.91 (0.84, 0.98)
Yang et al. 2021 [133]	Gene expression	CCT	CAGE eQTL + GTEx eQTL summarised data	2765 + 338	12 + 6SNPs	SMR	NA
Currant et al. 2021 [138]	IOP, RNFL GCIPL	POAG IOP	UKBB; IGGC	31,434	46 127 68	Bidirectional two-sample MR	IOP-POAG: 0.55 (2.80E-85) GCIPL and RNFL meta-POAG: -0.22 (0.10) POAG-GCIPL: -0.16 (0.09) et.al
Zhong et al. 2021 [7]	Circulating 25(OH)D levels	Behçet	Chinese cohort Turkish cohort	999 + 1585; 1215 + 1278	4	MR-IVW	3.96 (1.72, 9.13)
Zhong et al. 2021 [164]	Tuberculosis	Behçet	Chinese cohort Japan cohort	999 + 1585 + 4417; 611 + 737	5	Two-sample MR	1.26 (1.12, 1.43)
Bonelli et al. 2021 [167]	Metabolites, T2D and retinal vasculature	MacTel	23 participating clinical centres	476 + 17,33	mPRS; tPRS	Two-sample MR	Serine: 0.52 (0.46, 0.58)
Bonelli et al. 2021 [168]	Metabolites; retinal vascular calibre; T2D; macular thickness	MacTel	23 participating clinical centres	1067 + 3799	PRS	Two-sample MR	Serine:0.55 Glycine:0.81 Alanine:1.16

*AGEN* Asian Genetic Epidemiology Network, *AL* axial length, *AMD* age related macular Degeneration; APN, Adiponectin, *AREDS* Age-Related Eye Disease Study, *BMES* Blue Mountains Eye Study, *BMI* body mass index, *BW* birth weight, *CCHS* Copenhagen City Heart Study, *CCT* central corneal thickness, *CFH* complement factor H, *CH* corneal hysteresis, *CGPS* Copenhagen General Population Study, *CMUH* China Medical University Hospital, *CR* corneal radius of curvature, *CRAE* central retinal arteriolar equivalent, *CRVE* central retinal venular equivalent, *CREAM* Consortium for Refractive Error And Myopia, *CRF* corneal resistance factor, *CPR* C-reactive protein, *DBP* diastolic BP, *DR* Diabetic retinopathy, *GAMA* Genetics of AMD in Asian, *GCIPL* ganglion cell inner plexiform layer, *GERA* Genetic Epidemiology Research Study on Adult Health and Aging, *GLGC* Global Lipids Genetic Consortium, *HDL-C* High density lipoprotein cholesterol, *HDL-3* High-density lipoprotein 3 cholesterol, *IAMDGC* International AMD Genomics Consortium, *IGGC* International Glaucoma Genetics Consortium, *LDL-C* Low-Density Lipoprotein Cholesterol, *MAGIC* Meta-Analyses of Glucose and Insulin-Related Traits Consortium, *IOP* Intraocular pressure, *POAG* Primary open angle glaucoma, *IOPcc* corneal-compensated intraocular pressure, *KORA* Kooperative Ge-sundheitsforschung in der Region Augsburg, *MAP* mean arterial pressure, *TWB* Taiwan Biobank, *UKBB* UK Biobank, *PGIMER* Post Graduate Institute of Medical Education and Research, *PRS* polygenic risk score, *PSC* Posterior Subcapsular Cataract, *TG* triglyceride, *RE* Refractive error, *RNFL* retinal nerve fibre layer, *RRD* retinal detachment, *SBP* systolic BP, *SEED* Singapore Epidemiology of Eye Disease study, *SMR* Summary data-based MR, *SSGAC* Social Science Genetic Association Consortium;T2D,Type 2 diabetes, *TSLS* Two-stage least squares approach, *mPRS* metabolic polygenic risk scores, *tPRS* traits polygenic risk scores, *GRS* genetic risk score, *MacTel* Macular telangiectasia type 2, *EPIC* European Prospective Investigation into Cancer study, *eQTL* expression quantitative trait loci, *MR-IVW* MR Inverse-Variance Weighted, *CI* confidence interval, *OR* odds ratio, *NA* not applicable

### Age-related cataracts and obesity

Age-related cataracts are defined by opacity of the lens due to lost optical clarity concomitant with ageing, and are common causes of vision loss worldwide [16]. Energy and nutrient intake are involved in cataract formation and progression [17]. Several epidemiological studies have also identified a link between obesity and cataracts [18, 19]. An Asian population study used MR analysis to identify causal associations between fat mass, the obesity-associated gene (*FTO)* and various types of senile cataracts [20]. Accordingly, no causal associations were identified between obesity and cortical or posterior subcapsular (PSC) cataracts, whereas *FTO* was putatively involved in nuclear cataract pathogenesis. However, this study did not test the three key aforementioned MR assumptions. It is noteworthy that genetic variations at the *FTO* locus are associated with lipid metabolism pathways. Thus, further studies are warranted to investigate if lipid metabolism has a potential role in cataract pathogenesis. In another study, a two-stage MR approach was used to process Blue Mountains Eye Study data, and identified the single nucleotide polymorphism (SNP), rs9939609 as strongly related to exposure (obesity); the SNP was used as an IV to analyse the causal relationship between obesity and age-related cataracts [21]. Furthermore, different regression models were applied to verify the MR hypothesis (assumption i and ii). Interactions between the *FTO* SNP and protein intake were shown to increase the risk of PSC cataracts in individuals with dyslipidemia. However, these studies reflected a common issue with MR: no method has yet been identified to verify the existence of horizontal pleiotropy.

### Age-related macular degeneration (AMD) and related risk factors

Globally, AMD is a leading cause of blindness in the elderly. Several studies have proposed that particular genes are involved in AMD pathogenesis and may participate in inflammatory and complement pathways [22–24]. The Y402H SNP in the complement factor H gene, *CFH* is associated with AMD [25–27]. Increased *CFH* expression was observed in vitro in cultured (dedifferentiated) human retinal pigment epithelium (RPE) cells by immunohistochemistry [27]. *CFH* variations may lead to the improper control of the local complement system which triggers drusen formation [25]. Based on this evidence, a North Indian population-based study used MR on *CFH rs1061170* as an IV to demonstrate the polymorphism affected AMD occurrence or progression by modulating serum CFH levels [28]. From their findings, *CFH (rs1061170)* C allele carriers had an increased risk of AMD due to decreasing serum CFH levels. Proteomic analyses subsequently confirmed that some major proteins (e.g., C-reactive protein, (CRP)) occur in drusen as hallmark lipid and proteinaceous deposits [29]. Furthermore, to assess the causal relationship between circulating CRP levels and AMD, Han et al. selected SNPs associated with serum CRP levels as IVs for two-sample MR analysis [30]. They performed IVW analysis, then multivariable MR analysis which regressed several potential AMD factors based on univariate analysis. Combined, these authors showed that inflammatory pathways were involved in AMD pathogenesis, and that CRP was causally related to AMD. Unfortunately, this study did not use the non-linear IV hypothesis to test non-linear relationships between circulating CRP levels and AMD. Thus, further investigations are required to manage non-linearity in similar MR analyses.

In clinical epidemiology, AMD is commonly classified into early, intermediate and late stages. Late-AMD stages often lead to visual impairment and even irreversible loss of central vision [31, 32], and its treatment modality is different to other AMD stages. In early and intermediate AMD stages, pigmentary abnormalities and extracellular aggregate (drusen) accumulation occurs in the macular region [24, 33]. Moreover, lipid is a structural component of drusen [34]. Several observational studies explored relationships between lipid profiles and AMD risk and concluded that high levels of high-density lipoprotein cholesterol (HDL-C) increased disease risk [35–38]. However, owing to different study designs and sample sizes, potential confounding factors and reverse causality cannot be excluded, thus conclusions remain largely inconsistent [39, 40].

Importantly, GWAS have been instrumental in identifying associations between AMD and some genetic variants, such as hepatic lipase (*LIPC*) [41], cholesterol ester transfer protein (*CETP*) [42] and apolipoprotein E (*APOE*) [43]. Subsequently, several studies have exploited these genes to explore causal effects in different lipid pathways in AMD [44–46]. A previous two-sample MR study conducted in European ancestry individuals from the International Age-Related Macular Degeneration Genomics Consortium, involving 185 lipid-related genetic variants as IVs, identified a causal relationship between HDL-C and AMD, with an odds ratio (OR) of 1.22 [47]. In fact, some of the lipid deposition associated with AMD comes from the induction of the retina. Thus far, no validated IVs have been identified for retina lipids. Additionally, a multi-ethnic genetic study using three different MR models showed that HDL-C was a causal factor for advanced AMD [48]. Both studies used multivariable MR analyses which advantageously adjusted pleiotropy caused by incomplete independence between genetic variations and tested exposure variables.

Due to the huge variety of lipids and different AMD stages, specific research on relationships between lipid subcomponents and early and intermediate AMD stages is lacking. Recently, a large-scale study using a two-sample MR approach investigated the causal effects of eight major serum lipid biomarkers toward different AMD subtypes [6].Using a multivariable MR method based on Bayesian model averaging applied to exposure factors, the study identified lipid metabolism (apolipoprotein A1 and apolipoprotein B) functions in drusen formation, in particular at early and intermediate AMD development stages. MR approach based on Bayesian model averaging (MR-BMA) is a new modality for multivariate MR that incorporates more lipid particles using high-throughput experiments. Verena Zuber et al. used this method to prioritize risk factors by marginal inclusion probability (MIP), and then repeated analysis by modifying a posteriori probability, finally verified the strong causal effect of HDL on AMD, and also found the causal relationship between large and extra-large HDL particles and AMD [49]. The verification idea of MR-MBA is also superior to the IVW method of biased estimation of risk factors. It believes that the risk factors that really play a causal role themselves are a minority of a large number of risk factors, and more risk causal factors can be found through screening and sorting.

Apart from lipid profiles, a previous systematic review summarised several other potential risk factors for AMD [50] and showed inconsistent associations between refractive error (RE) and disease [51, 52]. Wood et al. conducted a two-sample MR study with IVW, and selected 126 genetic variants associated with RE as IVs to verify if RE was a risk factor for AMD in individuals of European descent [53]. Their analysis showed that hyperopia had a minimally positive causality with AMD, with an OR = 1.08. In addition, their sensitivity analyses, after excluding 31 variants closely related to non-RE traits, generated similar results (OR = 1.069). These inconsistent associations between RE and AMD may be due to non-causal factors such as parametric variations, stochastic fluctuations, potential confounders or selection bias.

### RE and related factors

RE is associated with many eye abnormalities and is one of the most common causes of visual dysfunction [54] For example, the pathological consequences of myopia, including exudative myopic macular degeneration and rhegmatogenous retinal detachment (RRD), are common causes of irreversible blindness [55]. Conventionally, observational epidemiological studies have postulated associations between some lifestyle exposures and myopia. For instance, education level was commonly regarded as a social factor leading to myopia onset, however, it is unclear if the relationship is causal [56]. Therefore, Cuellar-Partida et al*.* used GWAS education polygenic risk scores/data from the Social Science Genetic Association Consortium as IVs, and used a two-stage least squares approach to explore causal relationships [57]. Their analyses suggested a causal relationship existed between education and myopia, with estimated effect sizes higher than observational study estimates. Above findings have advantages in terms of strengthening due to the mediating variables that may confuse causality mentioned in the previous article were also excluded in the process of MR analysis [58, 59]. However, the other side of this causal relationship may be overestimated due to the existence of other education-associated markers (e.g., time spent on study). Therefore, more studies are required to investigate causal relationships between education and myopia, adjusted for more potential confounders.

To fully understand causal links between education and myopia, a bidirectional MR study based on the UK Biobank used a specific set of seven different assumptions and suggested exposure to more years in education increased myopia risk [60]. Two analyses in bidirectional MR, only one proved the causal relationship between the two variables: the longer the education, the more likely to develop myopia. This study is significant for strengthening the myopia prevention because it replaced the traditional regression analysis to detect potential pleiotropy.

Despite evidence from observational studies showing associations between myopia and vitamin D levels, the causal evidence for vitamin D associations with myopia is lacking, and are independent of outdoors activity times [61] or other related factors (e.g., time spent at work or exposure to natural light) [62, 63]. In view of this, Cuellar-Partida et al. selected effect estimates from a large RE meta-analysis from the Consortium for Refractive Error And Myopia (CREAM) and three other young cohorts to calculate causal estimates between vitamin D levels and myopia using Wald-type ratio estimators in an MR framework [64]. Valid and significant IVs identified by this study included 25-hydroxyvitamin D [25(OH)D] SNPs, as also determined by Afzal et al. [65] and Mokry et al. [66]. The meta-analysis identified no genetic causal links between 25(OH)D levels and myopia and further suggested associations were mediated by potential factors, including outdoor exercise times. Importantly, this analysis reflected a major MR benefit, i.e., unlike the temporary influence of exposure factors reflected by measured values in observational studies, MR IVs reflect the long-term exposure-induced state of the research factors.

In ophthalmologic anatomy, axial length (AL) and corneal radius of curvature (CR) are the two main physiological indicators of emmetropia. Previous studies reported that changes in children's growth trajectory could predict RE [67] and that correlations existed between birth parameters and ocular biometrics [68]. Tideman et al. conducted an MR analysis using weighted GRS as IVs and linear regression analysis to identify associations between prenatal and postnatal growth variables and AL and CR[69]. These authors showed that body growth parameters were significant predictors for a higher AL and corneal curvature (CC) by the age of six. The same genetic pathways between birth parameters and ocular biological indices were related to the study of causal associations between foetal growth, infancy, early childhood and REs.

Additionally, a low birth weight (LBW) is considered a common cause of visual impairment, and its relationship with RE has attracted considerable attention [70]. Some observational studies reported that LBW increased the risk of RE in school-age children (7–12 years) [71, 72], however, it was unclear if individuals with LBW within the normal birth weight range experienced RE in adulthood. Therefore, a one-sample MR analysis with GRS’s as IVs and a linear regression analysis was conducted, and showed that LBW within the normal range was causally associated with a more myopic RE, while the impact of causal effects was modest in UK Biobank participants [73]. In fact, race/ethnicity are factors associated with myopia; the myopia prevalence in East and South-East Asian is higher than European populations [55, 56]. Thus, future research must identify causal relationships between LBW and refractive changes, especially in Asian populations.

Currently, it is difficult to distinguish the effects of different environmental factors on RE, which are primarily due to heterogeneous exposure in the population. A causal role for particular medications in promoting RE was described long before MR research commenced [74]. For example, dorzolamide is a sulfonamide drug and was previously linked to transient myopia episodes [75, 76], whereas the same conclusion was drawn from a recent MR study by Patasova et al. [77]. Due to inevitable correlations between some medications and primary disease, Patasova et al. used SNPs associated with primary diseases as IVs and their effects over spherical equivalent (SE) for the outcome and suggested a causal effect of multisite chronic pain over hyperopic RE 74. One of the underlying causes of hyperalgesia is the lower threshold of sensory neuron firing, which may be related to genetic variations affecting proton-gated cation channels [78]. Likewise, RE is related to genetic polymorphisms in deionised cation channels [79]. Moreover, chronic pain may activate autonomic responses leading to pupillary dilation and accommodation relaxation [80]. Interestingly, this latter MR study showed that higher intraocular pressure (IOP) caused a greater myopic degree (MR-Egger *P* = 0.02), but without directional pleiotropy (intercept *P* = 0.256). Notably, this relationship was not mediated by IOP-lowering medication [81]. Furthermore, researchers observed that oral anti-diabetes drugs (metformin or gliburonide) may directly cause RE (MR-Egger *P* = 0.003, intercept *P* = 0.166). These findings suggest the effects of drugs on the ocular system must be accounted for when treating specific diseases.

### RRD and IOP myopia

RRD is one of the main emergency indications in ophthalmology and is the most common retinological emergency threatening vision [82, 83]. Several studies reported that when compared with non-myopes, individuals with greater changes in SE were at a greater risk of RRD and suggesting myopia may be a risk factor for RRD [84, 85]. Surgery is one of most common treatments for RRD, however, IOP may change after this surgery [84, 85]. Han et al. conducted a two-sample MR analysis using the GWAS dataset from the UK Biobank cohort and identified a causality between myopia, IOP and the risk of RRD [86]. In the same study, the effect of mean SE on RRD was greater than IOP, whereas a weak correlation was identified between mean SE and IOP. Furthermore, in subsequent sensitivity analyses, a stable causal effect of IOP on RRD was verified by excluding SE loci from IOP genetic structures using multivariable MR analysis. Myopia and IOP were causally associated with the risk of RRD, thus, myopia prevention efforts could help prevent disease. In the future, specific measures must be evaluated to prevent myopia during RRD, such as using atropine [87] and increasing outdoor activities [88].

### Glaucoma and related risk factors

Glaucoma is a common leading cause of irreversible blindness; by 2040, it is estimated that approximately 118 million people will suffer with the condition [89]. Progressive retinal ganglion cell degeneration and unique visual field-loss are the main clinical manifestations of primary open-angle glaucoma (POAG) [90]. Unfortunately, due to asymptomatic glaucoma, deriving a correct diagnosis at early disease stages in many patients is difficult [91]. The 2013 US Preventative Services Task Force recommendation statement reported that IOP was a modifiable risk factor in patients with POAG; its decrease prevented disease progression and emphasised the importance of an early diagnosis and treatment [92]. Notably, 10%–20% of POAG patients have normal or low IOP or "normal tension glaucoma" [93]. Therefore, glaucoma control measures cannot use IOP as the only indicator; in fact, many other factors are associated with glaucoma, such as hyperglycaemia. To the best of our knowledge, diabetes cannot only cause micro-vascular lesion-diabetic retinopathy, but may be associated with glaucoma [94, 95], however, epidemiological and clinical studies have not established a causal link between these conditions [96, 97]. Most studies, using MR analysis, have focussed on relationships between POAG and type 2 diabetes (T2D) [94, 95]. Previous studies screened relevant SNPs from an external GWAS database [98–100], calculated different genetic risk indices (overall genetic risk index and mechanism-specific genetic risk indices) as IVs, and confirmed causality between T2D and POAG using mechanism-specific genetic IV analyses. Furthermore, these authors emphasised the key role of pancreatic β-cell functions in POAG. Since T2D is a binary variable, this conclusion suggested T2D patients with metabolic disorders caused by pancreatic β-cell dysfunction would have a higher glaucoma risk. These studies also reported that the pleiotropic melatonin receptor 1B gene (*MTNR1B*) may have directly affected POAG via other mechanisms, independent of T2D. Accordingly, two loci (rs7708285 and rs6943153) were nominally significantly associated with POAG via fasting blood glucose, but the direction of effect was opposite. Thus, more studies on causal associations between fasting blood glucose and POAG, and the role of *MTNR1B* in POAG susceptibility are warranted.

Apart from diabetes, relationships between other metabolic disorders and IOP have been expounded [101, 102]. Hysi et al. reviewed the impact of different factors on IOP using metabolomics, Random Forest machine learning algorithms, and MR-IVW analyses to explore relationships between circulating metabolites and IOP [103]. From three MR models, O-methylascorbate had a significant IOP-lowering effect. Specifically, raising the levels of this metabolite reduced IOP causally, while IOP had no causal effect on O-methylascorbic levels. O-methylascorbate is a circulating metabolite of vitamin C, which is an antioxidant vitamin. In future research, it will be important to extrapolate if vitamin C alters IOP through antioxidant stress mechanisms [104].

Hyperlipidaemia is also a widely studied POAG biomarker [105, 106]. A two-sample MR analysis using GWAS summary data as IVs, involving three plasma lipid components (low density lipoprotein cholesterol [LDL-C], HDL-C and triglyceride (TG)) as exposure variables, showed no significant causal relationships between hyperlipidaemia and POAG risk [107]. However, only a causal association between routine lipid components and POAG was identified [107]. In fact, several other lipoprotein particles defined by the internal lipid content affected the biological function of lipid metabolites [108, 109]. Nusinovici et al., using a Bayesian network analysis, showed that total HDL-C was directly associated with POAG [110]. SNPs that correlated with HDL-C were then selected as IVs for MR-IVW, weighted median, mode based estimator and configuration mixture (Remix) method analyses. The findings suggested a causal and specific relationship between HDL-C and POAG. Mechanistically, small, dense HDL sub-fractions could mediate the cholesterol outflow pathway via the ATP-binding cassette 1 transporter (ABCA1) pathway [111, 112]. Also, imbalanced cholesterol levels can affect neurodegenerative disease [113]. High cholesterol-24-hydroxylase levels were identified in the retinas of glaucoma patients which may have damaged optic ganglion cells [114]. Additionally, HDL could contribute to POAG by influencing choroidal blood perfusion processes [115]. Therefore, future functional studies on different lipid particles must examine if these molecules participate in glaucoma pathological mechanisms by affecting blood perfusion.

Corneal hysteresis (*CH*) and corneal resistance factor (*CRF*) are both quantitative genetic indicators of corneal biomechanics. *CH* is a dynamic marker reflecting corneal structural and functional changes associated with IOP [116, 117]. CRF is calculated using a coefficient that emphasizes the first applanation. These biomechanical properties affect corneal-compensated IOP (IOPcc) measurements, which are used as estimated IOP values [118, 119]. Both indicators are genetically associated with ocular phenotypes and disease [120–122]. Simcoe et al*.* identified more than 200 loci related to corneal biomechanical properties using GWAS and the UK Biobank database, and conducted MR analysis to prove causal relationships between corneal biomechanics and IOP, and the directions of effect showed increased IOPcc reduced CH while simultaneously raising CRF [123]. This study also identified an association between genetically induced *CRF* and POAG, thus, future work could explore if the effects of *CRF* on POAG are driven by IOP.

A previous study reported that acute IOP increases could be associated with caffeine intake [124]. Although no links were identified between habitual caffeine intake and IOP changes in healthy subjects, increased caffeine consumption was associated with IOP increases in subjects with increased IOP susceptibility [125]. Similarly, consistent outcomes were identified in individuals with a family history of glaucoma [126]. Currently, causal evidence is lacking on whether reductions in long-term caffeine intake can control IOP and reduce glaucoma risks. Kim et al. conducted a two-sample MR analysis (UK Biobank database) using eight SNPs, which were significantly related to caffeine intake as IVs, to assess causal relationships between intake and IOP. Also, polygenic risk scores related to high IOP were calculated to test if genetically induced higher IOP altered associations between caffeine intake and IOP [127]. These authors observed a weak effect of long-term caffeine consumption on reducing IOP, but no causal relationship was identified. In contrast, in subjects with a strong genetic susceptibility to higher IOP, greater caffeine consumption would increase IOP and lead to higher glaucoma prevalence.

Another important corneal biomechanical indicator, central corneal thickness (CCT) shows strong heritability, with variability dependent on ethnicity [128, 129]. In a South African Eye Study, CCT in Africans was significantly lower than in other races [130]. Furthermore, a thinner CCT was identified as a risk factor for POAG [131]. Choquet et al. selected the main CCT related single nucleotide variants as IVs for a two-sample MR analysis, and showed a null causal association between thinner CCT and POAG [132]. From these results, the phenotypic effect of a single SNP may have been reduced by the data inclusion criteria, or other factors such as the environment or epigenetics may have contributed.

A SMR analysis was conducted in Asian and European populations; multiple genes, *CLIC3, ILMN_1796423, PTGDS, ILMN_1664464, C9orf142* and *ILMN_1761138* were identified and showed pleiotropic associations with CCT, but only in European ancestry participants [133]. Importantly, this study reflected the role of gene expression traits on outcomes in an MR framework. However, expression quantitative trait loci (eQTL) data were obtained from blood rather than eye tissue, and the same eQTL data were used in the analysis of different races. In the future, eQTL data must be sought from human eye tissue, and data analysis must be expanded to different races.

Optical coherence tomography (OCT) is an intravascular imaging technology widely used for the early diagnosis and management of ophthalmic diseases [134, 135]. Previous research showed that the thickness of the two inner retina layers, the retinal nerve fibre layer (RNFL) and ganglion cell inner plexiform layer (GCIPL), could change at different glaucoma stages, suggesting these indices could function as biomarkers for predicting glaucoma [136, 137]. A two-sample MR analysis using OCT images from 31,434 UK Biobank participants identified a relationship between morphological retinal phenotypes (RNFL and GCIPL) and POAG [138]. Because the causal relationship between IOP and POAG was clear, this study concluded the combination of time and IOP made the inner layer of retina thinner. Also, GWAS outcomes showed that three chromosomal loci, 1q42.3, 17q25.3 and 21q22.13 were related not only to eye pigmentation which caused foveal hypoplasia, but also eye colour [139]. These loci were also significantly related to visual acuity. Based on known genes, we can study associations between appropriate exposure factors related to them (such as changes in eye structure colour or the change force of skin and hair colour) and vision loss. OCT images combined with MR analyses could help unravel the biological function of different retina layers and provide insights on causal relationships between retinal morphology and eye disease.

### Diabetic retinopathy (DR), plasma lipid and adiponectin (APN) correlations

Globally, DR is one of the leading causes of blindness in working-age individuals [140], therefore, identifying and modulating risk factors is essential in preventing this condition. However, the underlying causal factors remain unclear. A previous study used GWAS summary data to investigate if lipid changes causally increased DR risk, using MR-IVW methods across three subgroups comprising all ethnicities, Caucasians and Chinese [141]. No clear evidence suggested dyslipidaemia increased the risk of DR. However, an analysis of 12 pleiotropic TG-associated SNPs in the model suggested IVs could be associated with other lipid components. Three rs12678919, rs174546 and rs964184 SNPs were closely associated with the proliferator-activated receptor-α (*PPAR-α*) gene which is used to treat DR by reducing TG levels [142]. These observations suggested TGs may be involved in DR formation. However, as study data comprised summarised statistical data from large GWAS studies, no accurate blood lipid values were identified, therefore extrapolations to clinical settings are limited.

A correlation was previously identified between APN and DR. A previous meta-analysis showed APN levels were significantly associated with DR in patients with T2D [143]. An observational cohort study also examined if elevated APN levels accelerated retinopathy progression in patients with T2D, and suggested APN could be a biomarker for DR pathogenesis [144]. APN is an adipokine secreted by fat cells and has a variety of functions, including promoting blood vessel formation [145] and endothelial cell inflammatory responses [146]. To comprehensively ascertain if APN played an essential role in DR pathogenesis, Huang et al. performed an MR-IVW analysis on three SNP groups (GRS_ALL_, GRS_Limited_ and GRS_APN_) which were previously differentially correlated with APN levels, by setting different thresholds and calculating the GRS of each group as an IV [147]. The reason for choosing GRS: through the calculation of F statistic, it was found that there were numerous weak IVs among APN-associated SNPs. A causal association between APN and DR was not fully proven, as only data from IVs- GRS_ALL_ suggested elevated APN levels could causally increase DR risk. Interestingly, this causal relationship between APN and DR only focused on T2D. It was previously suggested that the effects of APN on inflammatory markers in type 1 diabetes (T1D) or T2D was opposite [148, 149]. Therefore, the effects of APN on DR in T1D patients must be comprehensively examined in future studies.

### Retinopathy, plasma lipid and glucose level correlations

Emanuelsson et al. used weighed allele combined scores from 11 gene variants related to LDL-C levels as IVs in a one-sample MR analysis to predict the causal impact of LDL-C on micro- and macro-vascular diseases, e.g., retinopathy [150]. Consistent with two-sample MR results, high LDL-C levels were not a causal risk factor for retinopathy and neuropathy, but a causal relationship existed between LDL-C, chronic kidney disease (CKD) and peripheral arterial disease (PAD) in the general population. The data showed LDL-C had different roles in both micro- and macro-vascular disease 147. Peripheral neuropathy is the most prevalent chronic diabetes complication and is based on sensory and autonomic nerve symptoms. Currently, treatments of peripheral neuropathy are limited to anti-diabetes drugs [151]; a previous meta-analysis reported these drugs could only improve diabetes development, with no apparent significant effects on neuropathy [152]. Therefore, it is important to ascertain if causality exists between glucose control and neuropathy. In other research, Emanuelsson et al. further explored the causal effects of glucose levels on micro- and macro-vascular disease (including retinopathy, nephropathy, CKD, neuropathy and PAD) [5]. Apart from PAD, they showed a causal relationship between glucose levels and micro- and macro-vascular disease.

### BD and related risk factors

BD is a chronic, multi-system disease of unknown aetiology [153, 154]. It causes pathological damage to various organs, comprising oral and genital ulcers and ocular and cutaneous lesions [155]. Although BD pathogenesis is unclear, genetic susceptibility, infection triggers and immune abnormalities have been identified as the main pathogenic mechanisms [156]. Previous studies reported that patients with BD could be treated by vitamin D supplementation to modulate immune and inflammatory responses [157–159]. Zhong et al. used allele scores formed by four variants related to serum 25(OH)D levels as IVs to show that higher serum 25(OH)D levels increased BD risk [7]. However, the study conclusions contrasted with the protective effects of vitamin D on BD in observational studies [158, 159]. Therefore, potential BD development must be monitored after high-dose or long-term vitamin D intake. BD is also characterised by typical clinical features such as uveitis [160], which has a prevalence of 38–714/100000 individuals [161]. Uveitis causes serious damage to the eye tissue and leads to eye complications, including cataracts, glaucoma and ocular tuberculosis [162]. Pathophysiologically, *Mycobacterium tuberculosis* causes inflammatory immune response in the body [163], but no evidence based on MR analyses have been reported. However, Zhong et al. conducted MR and *mycobacterium tuberculosis* T cell spot test (T-SPOT.TB) analyses and showed that tuberculosis infection increased the risk of several non-infectious uveitis conditions [164].

### Macular telangiectasia (MacTel) and metabolic pathways

MacTel is an uncommon ocular disorder potentially causing legal blindness, however, its pathogenesis is unclear [165]. From recent GWAS findings [166], Bonelli et al. performed an MR analysis and showed that serine depletion was the strongest causal driver of MacTel risk [167]. In addition, some MacTel-associated loci exerted protective roles toward retinal endophenotypes, suggesting similar pathogenetic pathways could exist between other eye diseases involved in retinal endophenotypes and MacTel. The study also identified a weak correlation between T2D and MacTel. Another larger-scale GWAS and MR analysis observed similar findings, with a significant causal effect of low serine levels on MacTel aetiology [168]. In addition, gene expression involving metabolic disorder in the aetiology of MacTel is more concentrated in RPE. More clinical studies must focus on how moderate serine levels could limit this condition.

### Retinal vascular calibre and blood pressure (BP)

Retinal arterial narrowing is associated with higher BP and ambulatory BP monitoring [169]. However, another study observed no correlation between BP and retinal venular calibre [170], while a population-based cross-sectional study showed that systolic blood pressure (SBP) was positively correlated with retinal venular diameter [171]. Li et al*.* performed a traditional multi-linear regression and MR analysis to explore correlations and causalities between different BPs and retinal vascular calibre [172]. From their findings, elevated diastolic blood pressure (DBP) and mean arterial pressure (MAP) displayed weak causal roles with arteriolar lumen diameter, but no causal relationship was identified between retinal venular calibre and BP levels. Since potential factors during pleiotropic testing studies were not comprehensive, further analyses are required to determine causal associations between other factors (blood glucose and blood lipids) and retinal venular diameter to prevent the occurrence and development of related chronic diseases.

### Opportunities for MR in ophthalmology

MR applies IV methods to observational data; IVs are genetic variants that provide reliable evidence inferring causal relationships between exposures and outcomes. Thus, MR has become instrumental in dissecting residual ophthalmology issues by combining genomics and other disciplines. Currently, large-scale GWAS have been used to identify gene loci related to eye traits and diseases, which have not only provided essential support for gene targeted therapies, but also identified preventable and modifiable environmental risk factors using MR. Indeed, specialised ocular trait measurement instruments are often required to identify apparent features in the eyes or physiological indices inside the eyeball. To measure eye traits that are not readily available, MR is a convenient tool as it analyses gene expression from blood tissue as IVs to identify exposure factors [133]. In addition, MR is also used to analyse the causal role of specific pathological pathways involved in common ocular diseases (e.g. how the pancreatic β-cell-pathway in T2D increases the risk of POAG [100]). These examples show how much underlying disease pathologies in ophthalmology have been clarified. With the development of technology, novel MR techniques have been applied to ophthalmology research, which can solve the directionality of causality and the interaction between phenotypes. In fact, there are three assumptions before implementing the MR method to make causal inferences between exposures and outcomes: 1. There is a causal relationship between exposures and outcomes; 2. There is no causal relationship between exposures and outcomes; 3. There is a bidirectional causal relationship between exposures and outcomes. When the basic MR method cannot fully explore these three situations, bidirectional MR becomes an auxiliary method to clarify the sequence of causality [173, 174]. A typical example in ophthalmology research is that education level is related to myopia. In a study using bidirectional MR, it is found that the causal relationship between them is unidirectional, and there is no reverse causality (the idea that myopia takes longer to learn is not valid[60]). In addition, the other advanced MR approach, multivariable MR analysis, which can be performed to identify associations between multiple categorical exposure factors with similar effects, allows genetic variants to be associated with more than one exposure in this analysis. At present, there are an increasing number of researches using multivariable MR analysis, which has played a unique advantage in the exploration of the etiology of mental illness and cardiovascular disease [175, 176]. The advantage of multivariable MR is that eliminating the exposure factors with strong causality and exploring the causality values of the exposure factors with relatively weak correlation (for example, IOP, which is weakly correlated with SE, has less influence on RRD than IOP, but IOP still has causal influence on RRD[86]).

### MR challenges in ophthalmology

Because so many eye diseases exist, and the visual damage caused by lesions in different positions of the eye are rarely the same, MR analysis can be used to identify unclear risk factors in ophthalmic diseases. Glaucoma is a severe ocular pathology and its most critical risk factor is IOP, however, this still does not explain the pathogenesis of all glaucoma types. In addition, it is unclear if substandard physiological indicators at birth (including height and weight) causally increase the risk of developing eye disease, therefore these indicators should be investigated. The exploration of causal factors with indispensable roles in cataractogenesis should not remain on obesity. Importantly, significant racial and ethnic differences impact on the generation of most eye diseases. However, gene identification presents two issues during MR analyses; in two-sample MR, public summary gene data from similar ethnic groups are not readily available. No large-scale GWAS have been conducted for some eye diseases, or genetic data are lacking for eye diseases across some races and ethnicities. Furthermore, as outlined (Table 2), the UK Biobank database is predominantly used in most studies and includes mainly European populations, the impact of sample overlap should be considered when discuss these studies. From our review, other databases exist; CREAM, the International AMD Genomics Consortium (IAMDGC) and the International Glaucoma Genetics Consortium (IGGC) and specialise in specific eye diseases. In addition, genetic data may not fully identify exposure factors to generate true causal associations. Genetic induction at the cell and molecular level is also worthy of investigation. For example, in addition to circulating lipids implicated in AMD, specific retinal effects on lipid pathways also affect AMD pathological mechanisms [47]. Although MR-Egger regression intercepts, pleiotropy residual sums, outlier global tests, and PhenoScanner have been used to assess pleiotropy or horizontal pleiotropy using IVs, wide-spread horizontal pleiotropy may potentially exist, which leads an important statistical challenge on assumptions being violated and biased statistical inference. Therefore, scientists must fully consider horizontal pleiotropy among IVs when drawing conclusions. At the same time, although the advanced MR approach can solve the phenotypic relationship with complex causality, there are also some problems in practice. When there is a bidirectional causality between exposures and outcomes, the difficulty in implementing bidirectional MR lies in the selection of reasonable genetic variants, as there will inevitably be an association between genetic variants associated with exposures and outcomes. Then, in order to avoid collinearity, multivariable MR also has requirements for the number of genetic variations: its number cannot exceed the number of exposure factors.

### Conclusion and future directions

In spite of the many promising applications of MR, the validity of causal findings depends on three key assumptions (i—iii) on valid IVs, which sometimes may not hold in practice. In fact, numerous open GWAS databases exist in different jurisdictions. Researchers can search for SNPs as IVs using GWAS catalogues and search for keywords related to the subjects to prove that they are not related to other traits, so as to satisfy the assumption [7]. From a methodology perspective, no clear and unified test method has yet indicated if pleiotropy IV’s exist in MR. The interpretation of MR results requires the consideration of numerous factors, including study design, appropriate IVs and biological complexity. A deep understanding of gene-environment interactions using BioBank genome-wide data on genetics, demographics, clinical factors and lifestyle habits may help triangulate with ophthalmic MR studies. Given the rapidly evolving research landscape, the replication of early MR studies using larger-scale multicentre investigations and improved genetic instruments continues to be of value.

In short, MR has improved ophthalmology research and helped researchers prevent and treat ophthalmic diseases. Equally, MR studies continue to provide strong evidence showing causal inferences between exposure factors and outcomes, which may guide future clinical trials and drug development, and provide a theoretical basis for clinical and public health decision-making.

## Supplementary Information

**Additional file 1.** The design framework of MR.

## Data Availability

There is no new data generated as part of this review.
