# Supplementary material for: Mendelian randomization analyses in ocular disease: a powerful approach to causal inference with human genetic data
Source: J Transl Med. 2022 Dec 26;20:621. doi: 10.1186/s12967-022-03822-9 (PMC9793675; doi:10.1186/s12967-022-03822-9)
Supplement: Supplementary file 1 — Additional file 1. The design framework of MR. [file 12967_2022_3822_MOESM1_ESM.docx]

**Additional file 1**

**

**

**Figure S1. Directed Acyclic Graph (DAG) of instrumental variables in causal associations.**

To explore the real relationship between exposure and outcome, genetic variations reflecting different degrees of exposure are used as instrumental variables (IVs) to evaluate causality. There are three IV assumptions:

i). the variant is associated with the exposure,

ii). the variant is not associated with any confounder of the exposure-outcome association and

iii). the variant does not affect the outcome, except possibly via its association with the exposure.


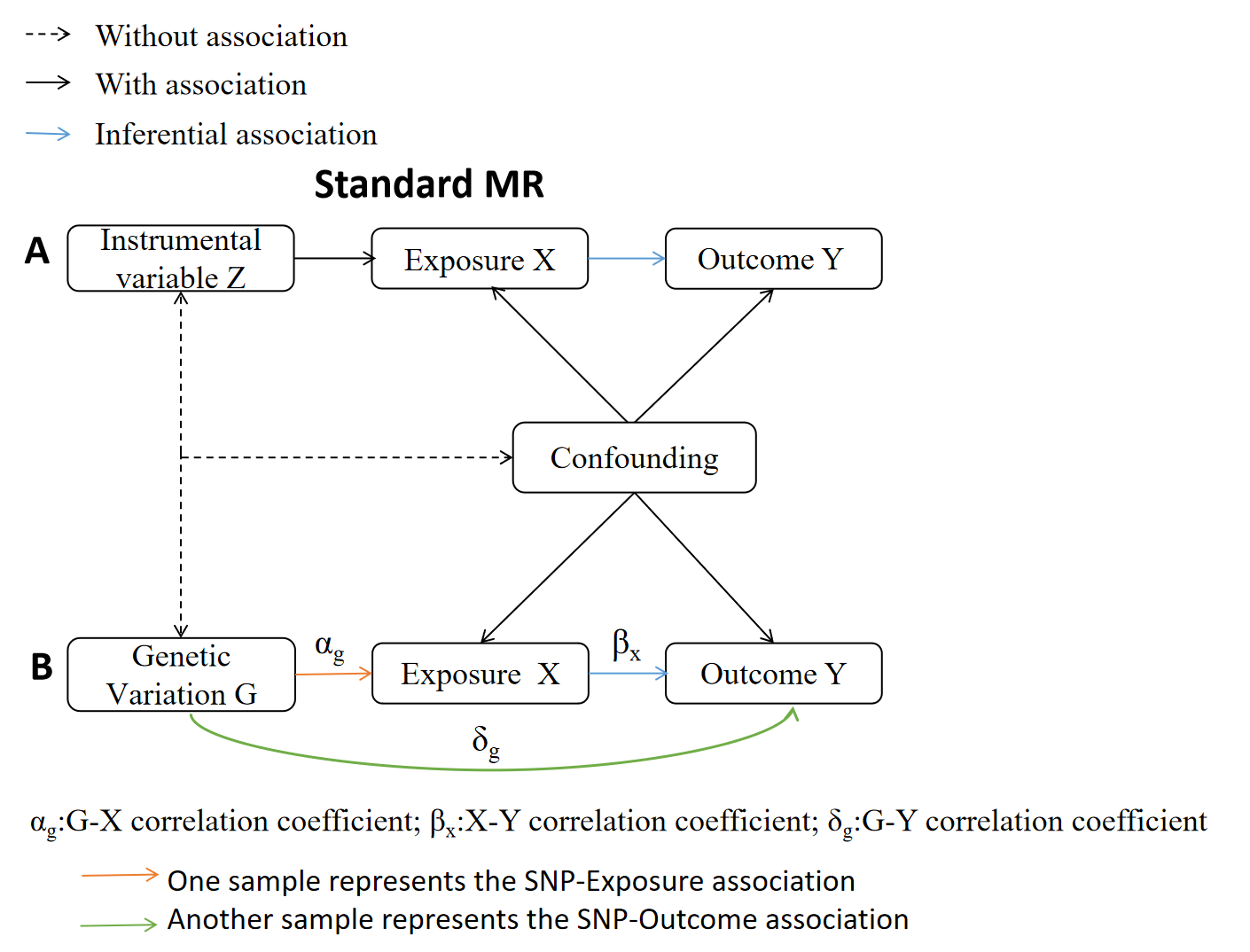


**Figure S2. A standard Mendelian Randomization framework**

1. One stage MR: This simply uses G-X and G-Y associations to directly infer the causality of X-Y.
2. One-sample and two-sample MR: One-sample MR is performed on a single study population using a two-stage least-squares regression (2-SLS) model that effectively infers causal effect values for X-Y. Two-sample MR uses the same principle as one-sample MR, but it evaluates single nucleotide polymorphism (SNP) associations with exposures and outcomes from two independent and unrelated samples.


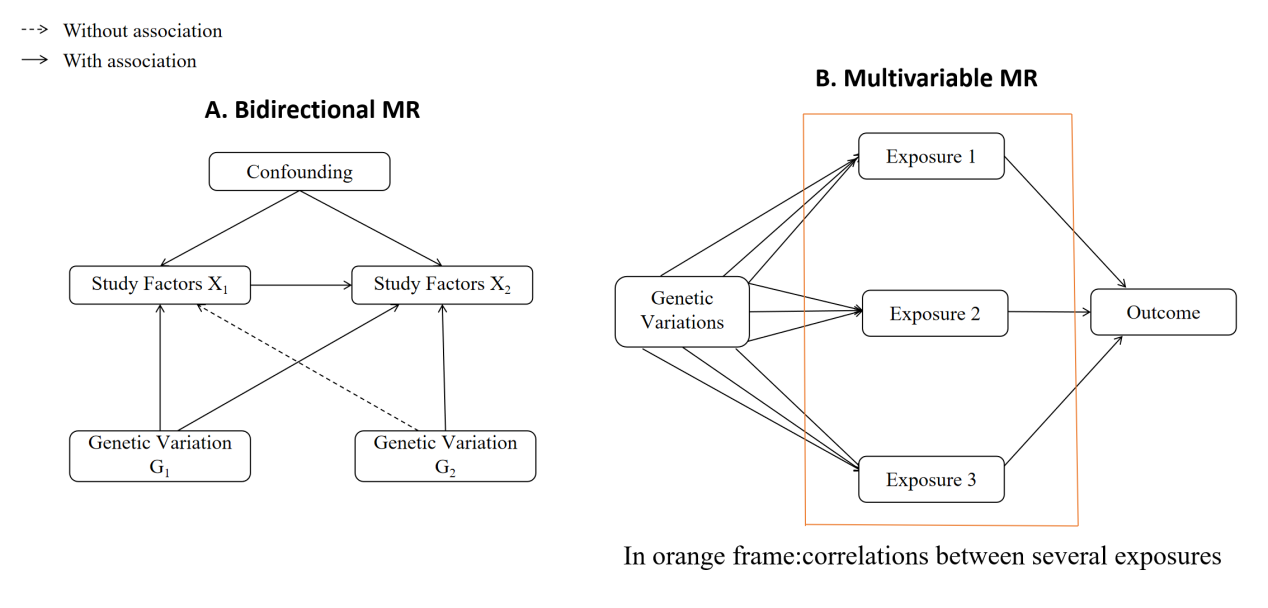


**Figure S3. Advanced Mendelian Randomization (MR) frameworks.**

1. Bidirectional MR: this approach untangles the direction of causal effect of factors and outcomes.
2. Multivariable MR: this approach is commonly used when single nucleotide polymorphisms (SNPs) are associated with more than one exposure.
